# Energy metabolism disorder dictates chronic hypoxia damage in heart defect with tetralogy of fallot

**DOI:** 10.3389/fcvm.2022.1096664

**Published:** 2023-01-18

**Authors:** Libao Liu, Lei Huang, Lishuai Yao, Fan Zou, Jinyuan He, Xiaodong Zhao, Lugang Mei, Shuai Huang

**Affiliations:** ^1^Department of Cardiothoracic Surgery, The Third Affiliated Hospital of Sun Yat-sen University, Guangzhou, China; ^2^Department of Gastroenterology and Rheumatology Immunology, The Third Affiliated Hospital of Sun Yat-sen University, Guangzhou, China; ^3^Heyuan Maternal and Child Health Hospital, Heyuan, Guangdong, China; ^4^Guangdong Provincial Key Laboratory of Research in Structural Birth Defect Disease, Guangzhou Women and Children’s Medical Center, Guangzhou Medical University, Guangzhou, China; ^5^Department of Cardiovascular Surgery, Zhujiang Hospital, Southern Medical University, Guangzhou, China

**Keywords:** tetralogy of fallot, congenital heart disease, metabolomic analysis, energy metabolism, liquid chromatography tandem mass spectrometry

## Abstract

**Background:**

Tetralogy of Fallot (TOF) belongs to cyanotic heart damage, which is the most common in clinic. In the chronic myocardial hypoxia injury related to TOF, the potential molecular mechanism of cardiac energy metabolism remains unclear.

**Materials and methods:**

In our study, microarray transcriptome analysis and metabonomics methods were used to explore the energy metabolism pathway during chronic hypoxia injury. The gene expression omnibus (GEO) dataset GSE132176 was obtained for analyzing the metabolic pathways. The clinical samples (right atrial tissues) of atrial septal defect (ASD) and TOF were analyzed by metabonomics. Next, we screened important pathways and important differential metabolites related to energy metabolism to explore the pathogenesis of TOF.

**Results:**

Gene set enrichment analysis (GSEA) indicated that fructose 6-phosphate metabolic process, triglyceride metabolic process, and et al. were significantly enriched. Gene set variation analysis (GSVA) results showed that significant difference of ASD group and TOF group existed in terpenoid metabolic process and positive regulation of triglyceride metabolic process. Pathways with significant enrichment (impact > 0.1) in TOF were caffeine metabolism (impact = 0.69), sphingolipid metabolism (impact = 0.46), glycerophospholipid metabolism (impact = 0.26), tryptophan metabolism (impact = 0.24), galactose metabolism (impact = 0.11). Pathways with significant enrichment (impact > 0.1) in ASD are caffeine metabolism (impact = 0.69), riboflavin metabolism (impact = 0.5), alanine, aspartate and glutamate metabolism (impact = 0.35), histidine metabolism (impact = 0.34) and et al.

**Conclusion:**

Disturbed energy metabolism occurs in patients with TOF or ASD, and further investigation was needed to further clarify mechanism.

## 1. Introduction

Tetralogy of Fallot (TOF) is a congenital cyanotic heart disease (CHD), the most common and fatal (1). TOF is considered as a prototype CHD (2), which affects 3 out of 10,000 live births (3). In addition, it is the most common cause of CHD (4), which represents that 7–10% of CHD occurs in 0.5/1,000 live births (5). TOF is mainly due to the abnormal development of the arterial cone, leading to pulmonary artery stenosis, ventricular septal defect, aortic span, and finally leading to right ventricular hypertrophy (6). The pathogenesis is very complex, which is closely associated with abnormal expression of some genes participated in formation of cardiovascular system in embryonic development and the dysfunction of related protein factors (7). The four features of the malformation are inferior, aortic coverage, and right ventricular (RV) hypertrophy (3). Chronic hypoxia stimulates the right ventricle in patients with TOF (8).

Children with TOF older than 2 months are at risk of postoperative myocardial systolic failure, and their metabolism is impaired, resulting in a decrease in the concentration of adenosine triphosphate (ATP) and an increase in the concentration of lactic acid (9). Myocardial hypoxia is one of the causes of TOF central ventricular dysfunction. Metabolic abnormalities during reperfusion indicate that mitochondrial dysfunction may lead to complications such as low output syndrome and low ventricular function (10). However, the effect of postnatal hypoxia on the pathogenesis of TOF is still unclear. Atrial septal defect (ASD) refers to the hypoplasia of the ventricular septum in the embryonic stage, which forms abnormal traffic and produces left to right shunts at the atrial level (11), as a most common CHD, accounting for 20∼30% of all congenital heart malformations (12). It is of great significance to screen potential differentially expressed metabolites in TOF and ASD myocardial tissues and explore the molecular mechanism related to TOF and ASD formation using the metabonomics technology.

In our study, bioinformatics and metabonomics analyses indicated that metabolites related to energy metabolism significantly changed in ASD and TOF. The metabolic pathways between pre-TOF and post-TOF and pathways between pre-ASD and post-ASD were analyzed. Finally, we identified important metabolic pathways in TOF and ASD.

## 2. Materials and methods

### 2.1. Data source

The dataset GSE132176 (13) and related information were obtained with Gene Expression Omnibus (GEO). In this study, right atrial (RA) samples from ASD and TOF patients before CPB were compared and analyzed. The original data of GSE132176 is downloaded. Then, “linear models for microarrays (LIMMA)” were applied to analyze the original data (14). Differentially expressed genes (DEGs) were analyzed conforming to Benjamin Hochberg method standard | log2 Fold Change (FC)| > 1.0 and adjusted *P* value < 0.05.

### 2.2. Gene ontology analysis

Gene ontology (GO) is a unified biological tool. In this paper, through “GOplot” R package, the GO function enrichment analysis was performed with the up-regulated DEGs and down-regulated DEGs to determine the biological process (BP), cellular component (CC) and molecular function (MF) items, with *P* value < 0.05.

### 2.3. Gene set enrichment analysis (GSEA) and gene set variation analysis (GSVA)

To determine whether there is a significant difference between 10 patients with TOF and 10 patients with ASD, GSEA was conducted (15, 16). Adjusted *P* value < 0.05 was defined as statistical significance. Collect genes with significance based on adjusted *P* value and | log2 FC| > 1 for subsequent analysis. With “GSVA” R package, the most relevant pathway were found (17). Adjusted *P* < 0.05 as significance. These gene sets are collected from the molecular signature database (MSigDB) in this script.^1^

### 2.4. Clinical samples

From June 1, 2018 to January 1, 2020, we collected 6 samples from the Third Affiliated Hospital of Sun Yat-sen University, involving 3 right artery (RA) biopsies of 3 TOF patients and 3 right artery (RA) biopsies of 3 ASD patients. Patient clinical characteristics were presented in Table 1. All research protocols of the study were approved by Ethics Committee of the Third Affiliated Hospital of Sun Yat-sen University (Approval number of the Ethics Committee: 202202-121-01). Informed consent of all patients and guardians has been obtained. All patients received a comprehensive physical examination. Clinical data, including medical records, electrocardiogram, echocardiography, and cardiac catheterization reports were reviewed systematically. The New York University Pediatric Heart Failure Index (NYU PHFI) and the modified ross score were used to assess cardiac function (18). Before cardiopulmonary bypass, RA myocardial tissue samples and serum were obtained from children with TOF or ASD after cardiac surgery. The collected tissue and plasma samples were frozen in liquid nitrogen and stored at −80°C immediately after resection.

**TABLE 1:** Clinical characteristics of patients.

Characteristics	TOF patients	ASD patients
**Age (Months)**		
≤ 10	1	0
> 10	2	3
**Gender**		
Female	1	1
Male	2	2
**Weight (kg)**		
≤ 7	0	2
> 7	3	1
**Cardiopulmonary bypass time (min)**		
≤ 60	0	1
> 60	3	2
**Hospital stay (Day)**		
≤ 10	1	1
> 10	2	2
**Mechanical support time (h)**		
≤ 10	1	2
> 10	2	1

### 2.5. Sample preparation and metabolite extraction

Extraction methods of hydrophilic substances: take out the tissues and blood on the tissue surface was sucked out with filter paper, then cut a sample weighing 50 ± 2 mg and transferred it to the centrifuge tube. Added a steel ball to the centrifuge tube, and homogenized 4 times at 30 HZ for 30 s each time. Added 1 mL 70% methanol internal standard solution (2-Chloro-Lphenylalanine) into the centrifuge tube and shook 5 min, then stood it on ice for 15 min. Sucked 400 uL of supernatant into new centrifuge tube after centrifuged at 4°C at 12,000 r/min for 10 min. Kept the supernatant in refrigerator at −20°C overnight and sucked 200 μL supernatant after centrifuged at 4°C and 12,000 r/min for 3 min. Put supernatant into injection bottle for LC-MS analysis.

Extraction method of hydrophobic substances: after the tissue was thawed, cut 20 mg of sample into a 2 ml centrifuge tube; added 1 mL of lipid extract (methyl tert butyl ether: methanol = 3:1, V/V, containing internal standard mixture), and added steel ball homogenate, then took out the steel ball and vortex for 15 min. Added 200 uL water and vortex for 1 min then centrifuged at 12,000 r/min and 4°C for 10 min; sucked 300 uL of supernatant into a 1.5 mL centrifuge tube, and concentrated at ultra-low temperature. Dissolved it with 200 uL mobile phase B for LC-MS/MS analysis.

### 2.6. Chromatography analysis

Liquid phase of hydrophilic substances was as follow:

Chromatographic column: Waters ACQUITY UPLC HSS T3 C18 1.8 μm, 2.1 mm × 100 mm; mobile phase: phase A was ultrapure water (0.1% formic acid), and phase B was acetonitrile (0.1% formic acid). Elution gradient was shown in Table 2.

**TABLE 2:** Elution conditions of hydraulic substances.

Time	Phase A (%)	Phase B (%)
0 min	95	5
11.0 min	10	90
12.0 min	10	90
12.1 min	95	5
14.0 min	95	5

Hydrophobic substance liquid phase condition was as follow:

Chromatographic column: Thermo Accucore™ C30 column, i.d. 2.1 × 100 mm, 2.6 um; mobile phase: Phase A was acetonitrile/water (60/40, V/V); Phase B was acetonitrile/isopropanol (10/90, V/V). Elution gradient was shown in Table 3. Injection volume was 2 μL.

**TABLE 3:** Elution conditions of hydrophobic substance.

Time	A (%)	B (%)
0 min	80	10
2 min	70	30
4 min	40	60
9 min	15	85
14 min	10	90
15.5 min	5	95
17.3 min	5	95
17.5 min	80	20
20 min	80	20

### 2.7. Mass spectrometry analysis

Mass spectrum analysis of hydrophilic substances: temperature of spray ionization (ESI) was 500°C, and set the mass spectrum voltage to 5,500 V (positive) and −4,500 V (negative), the ion source gas I (GS I) was 55 psi, the gas II (GS II) was 60 psi, the curtain gas was 25 psi.

Mass spectrum conditions of hydrophobic substances: the ESI was 500°C, the mass spectrum voltage in positive ion mode was 5,500 V, the mass spectrum voltage in negative ion mode was −4,500 V, the GS1 was 45 psi, GS2 was 55 psi.

### 2.8. Principal component analysis

Orthogonal partial least squares-discriminant analysis (OPLS-DA) and principal component analysis (PCA) were carried out using R (19). Metabolites with differential expression between two groups were identified by the variable importance in prediction (VIP) ≥ 1 (20) and | log2FC| ≥ 1 (4, 21). From the OPLS-DA results, we extracted VIPs, and we conducted permutation test (200 permutations) to avoid over-fitting.

### 2.9. Metabolic pathway analysis

With Human Metabolome Database,^2^ we annotated identified metabolites, then pathway analysis was conducted with MetaboAnalyst 5.0.^3^

## 3. Results

### 3.1. Identification of DEGs and GO analysis

**FIGURE 1:**
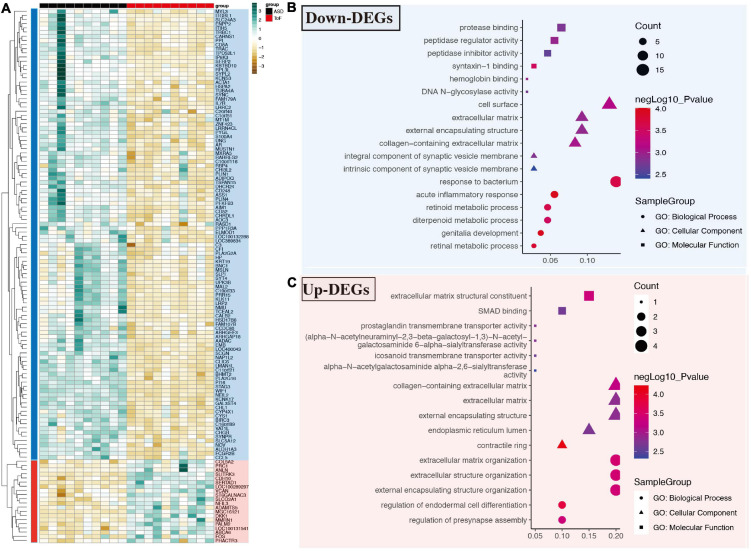
Differential expressed genes and gene ontology (GO) analysis in GSE132176 dataset. **(A)** Differentially expressed genes in tetralogy of fallot (TOF) vs. atrial septal defect (ASD). **(B)** GO analysis of down-regulated genes. **(C)** GO analysis of up-regulated genes.

Compared with the common samples, 128 differentially expressed genes were found in the TOF samples. These DEGs include 20 up-regulated and 108 down-regulated DEGs after data preprocessing. The DEGs expression level in each sample was shown in Figure 1A. In terms of GO analysis of down-regulated DEGs, the main MF items were protease binding, peptidase inhibitor activity, syntaxin-1 binding, hemoglobin binding, DNA N-glycosidase activity, and et al.; the main CC items were: cell surface, intrinsic components of synaptic vesicle membrane, and et al.; the top six BP items were response to bacterium, acute inflammatory reaction, retinoid metabolic process, diterpenoid metabolic process, genitalia development, and retinal metabolic process (Figure 1B). In terms of GO analysis of up-regulated DEGs, the top six MF items were extracellular matrix structural constituent, SMAD binding, prostaglandin transmembrane transporter activity, icosanoid transmembrane transporter activity, alpha-N-acetylgalactosaminide alpha-2,6-sialytransferase activity, and et al.; the main CC items were collagen-containing extracellular matrix, contractile ring, and et al.; the top five BP items were regulation of endodermal differentiation, regulation of presynapse assembly, and et al. (Figure 1C).

### 3.2. Result of GSEA and GSVA

**FIGURE 2:**
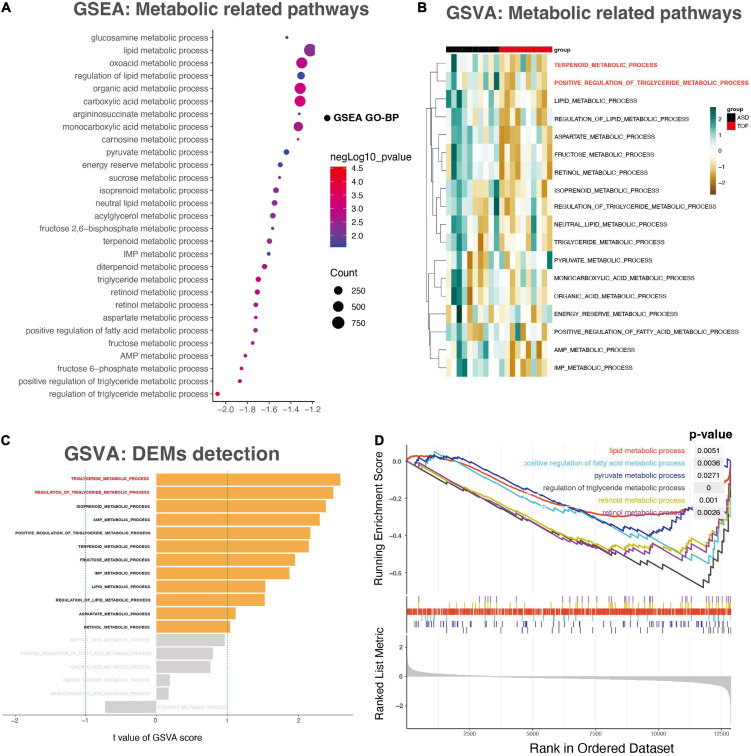
Gene set enrichment analysis (GSEA) and gene set variation analysis (GSVA). **(A)** The energy metabolism-related pathways detected through GSEA analysis. **(B)** The heatmap of the GSVA score for the KEGG pathway. **(C)** The bar plot for patterns of the GSVA score of energy metabolism. **(D)** The energy metabolism- related pathways detected through GSEA analysis.

**FIGURE 3:**
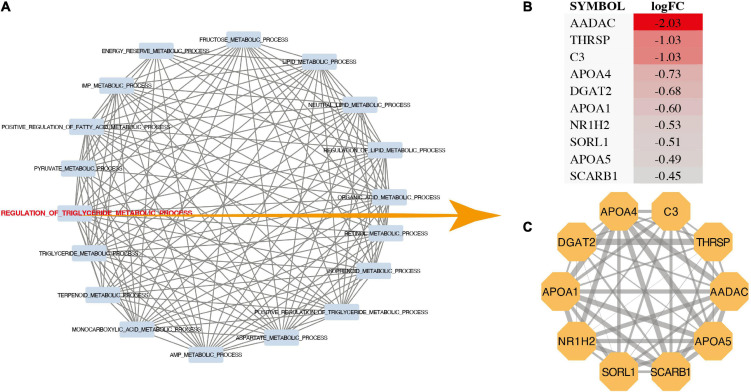
Pathway interaction analysis. **(A)** Pathway interworking network. **(B)** LogFC of genes in regulation of triglyceride metabolic process. **(C)** Protein-protein interactions network of genes in regulation of triglyceride metabolic process.

The results showed that the regulation of triglyceride metabolic process (*p* = 0.000028), carboxylic acid metabolic process (*p* = 0.00057), organic acid metabolic process (*p* = 0.00071), fructose 6-phosphate metabolic process (*p* = 0.00076), triglyceride metabolic process (*p* = 0.00094), retinoid metabolic process (*p* = 0.00097), oxoacid metabolic process (*p* = 0.00154), aspartate metabolic process (*p* = 0.00175), positive regulation of triglyceride metabolic process (*p* = 0.00192) were most significantly enriched (Figure 2A). In addition, the data set GSE132176 was used to verify the core genes. GSVA results showed that there was a significant difference between ASD group and TOF group in the positive regulation of triglyceride metabolic process and terpenoid metabolic process (Figures 2B, C). In addition, GSEA analysis showed that metabolic pathways have been identified. The results showed that lipid metabolic process (*p* = 0.0051), positive regulation of fatty acid metabolic process (*p* = 0.0036), pyruvate metabolic process (*p* = 0.027), regulation of triglyceride metabolic process (*p* = 0.000028), retinoid metabolic process (*p* = 0.001), and retinol metabolic process (*p* = 0.0026) were significantly identified (Figure 2D). The pathway interaction network was shown in Figure 3A. The interaction showed that regulation of triglyceride metabolic process was the most important way. Figure 3B showed the top ten genes with the highest FC in this pathway, namely AADAC, THRSP, C3, APOA4, DGAT2, APOA1, NR1H2, SORL1, APOA5, and SCARB1. The interaction network of these ten genes was shown in Figure 3C.

### 3.3. Multivariate statistical analysis

**FIGURE 4:**
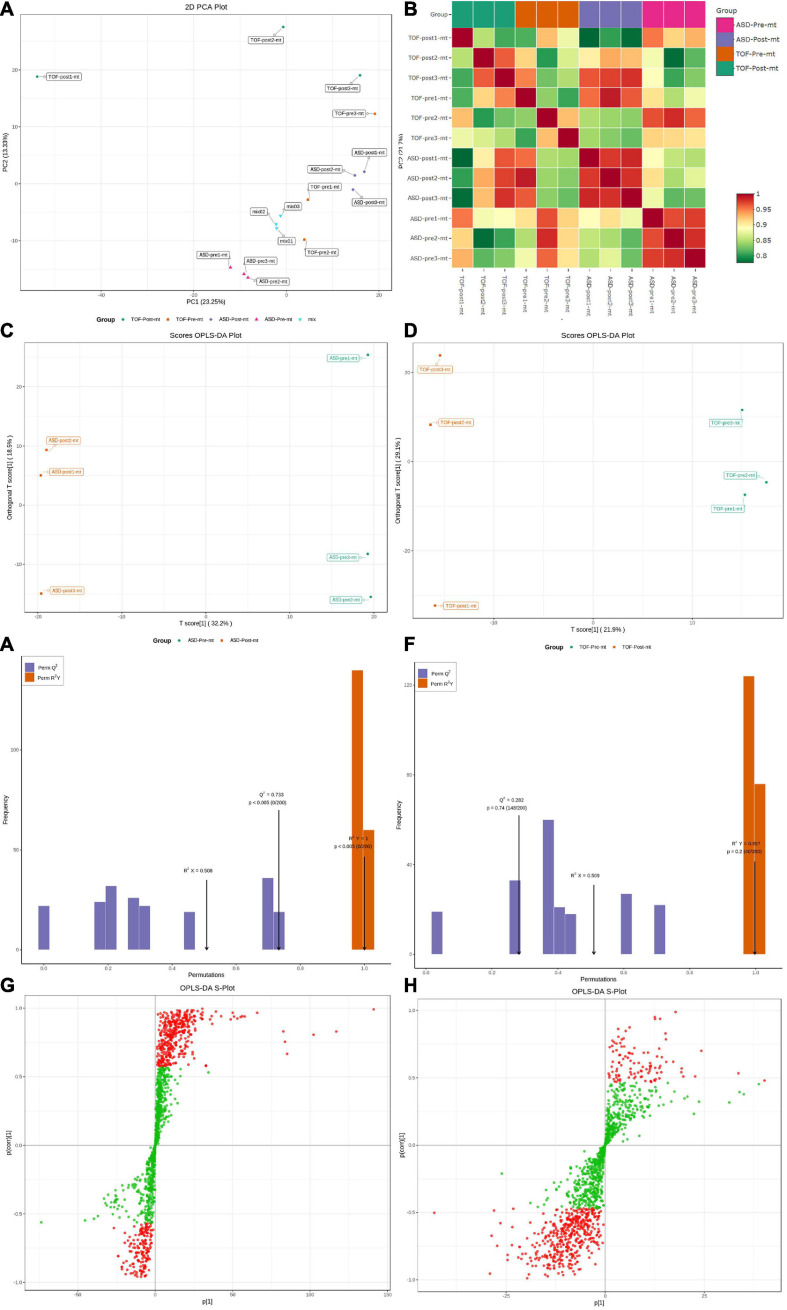
Principal component analysis (PCA) and orthogonal partial least squares-discriminant analysis (OPLS-DA) of atrial septal defect (ASD) and tetralogy of fallot (TOF). **(A)** PCA for all samples. **(B)** Heatmap of sample distances. **(C)** OPLS-DA for ASD samples. **(D)** OPLS-DA for TOF samples. **(E)** Fitting analysis of ASD samples. **(F)** Fitting analysis of TOF samples. **(G)** S-plot analysis of metabolites in ASD samples. **(H)** S-plot analysis of metabolites in TOF samples.

The PCA analysis (Figure 4A) showed that QC samples (mix01–mix03) were clustered together, which indicated that instruments were stable in long-term operation. The correlation analysis showed that correlation coefficient of most samples within the group was higher than that of samples between groups, indicating that the differential metabolites obtained were accurate and reliable (Figure 4B). Metabolites of each sample were screened using multivariate statistical analysis to obtain differences in the metabolic composition of the four groups of samples. OPLS-DA was used to identify differences and outliers between experimental groups pre-ASD and post-ASD, pre-TOF and post-TOF. The results showed that the groups pre-ASD and post-ASD, pre-TOF and post-TOF were obviously separated (Figures 4C, D). The OPLS-DA analysis indicated a valid model with *Q*^2^ > 0.05 (Figures 4E, F). Information of all metabolites and differentially expressed metabolites of TOF group and ASD group were shown in Supplementary Tables 1–3. Based on OPLS-DA model analysis, the metabolites which contribute the most to classification (VIP ≥ 1) were selected as important variables (Figures 4G, H).

### 3.4. Analysis of differential metabolites

**FIGURE 5:**
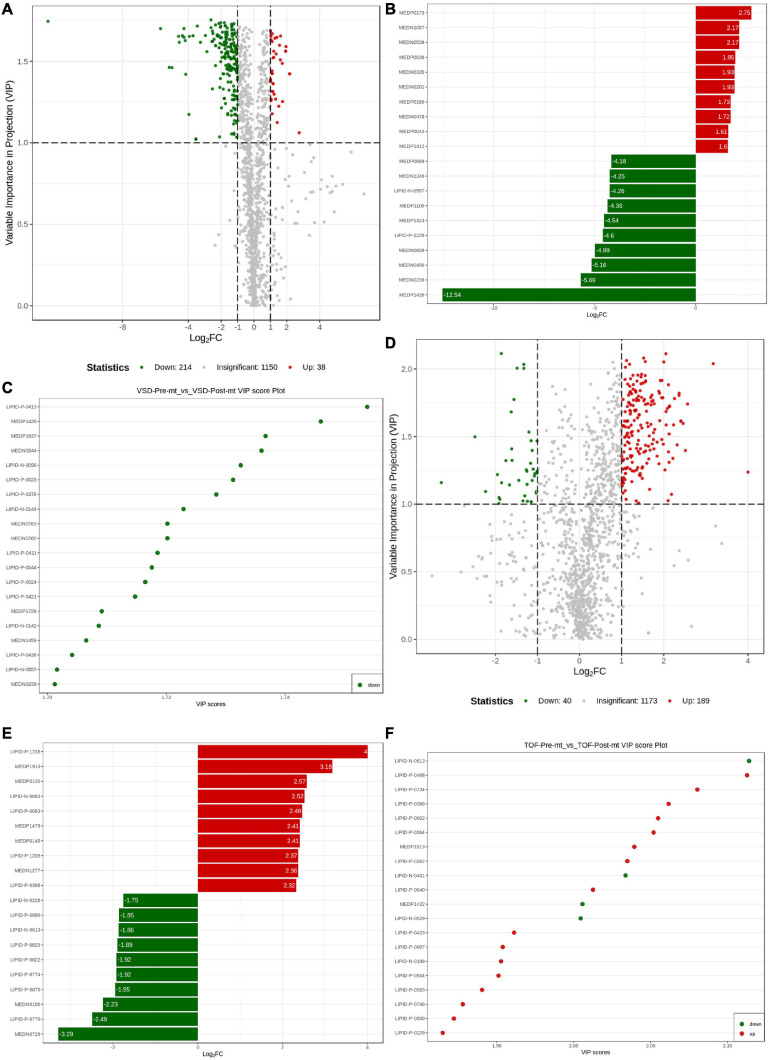
Differential metabolite analysis. **(A)** Volcanogram of differential metabolites in atrial septal defect (ASD) samples. **(B)** Up- and down-regulated of the top 10 metabolites in ASD samples. **(C)** Top 20 differential metabolites by VIP value in ASD samples. **(D)** Volcanogram of differential metabolites in tetralogy of fallot (TOF) samples. **(E)** Up- and down-regulated of the top 10 metabolites in TOF samples. **(F)** Top 20 differential metabolites by VIP in TOF samples.

VIP reflects each variable influence. Therefore, we first screened metabolites according to the VIP value of OPLS-DA. FC between pre-ASD and post-ASD groups were also used to screen differential expressed metabolites (DEMs). Finally, 252 endogenous metabolites with the largest contribution to the separation of post-ASD group and pre-ASD group were selected, 214 metabolites were down regulated, and 38 metabolites were up regulated (Figure 5A). According to MS/MS information and combined with online database, these metabolites were identified, and metabolites with large change rate were nicotinic acid adenine dinucleotide, UDP-D-galactose, UDP-glucose, 5-oxoproline, methylmalonic acid, succinic acid, β-Nicotinamide mononucleotide, aminomalonic acid, glutathione reducedform, carnitine C13:1, tranexamic acid, 3-(pyrazol-1-yl)-L-alanine, PI(20:4_20:4), 2-aminomethylpyrimidine, carnitine C7:DC, TG(16:0_20:4_22:6), uridine triphosphate, deoxycytidine, d-gluconic acid, carnitine C6:DC (Figure 5B). Figure 5C showed the significant DEMs with high VIP scores, including LPE(22:0/0:0), carnitine C6:DC, 11-dehydrocorticosterone, orotic acid, FFA(20:4), carnitine C18:0, LPC(22:1/0:0), LPE(20:0/0:0), (±)9-HETE, (±)5-HETE, LPE(18:0/0:0), carnitine C22:1, carnitine C20:0, LPE(22:1/0:0), 3-hydroxy-4-aminopyridine, LPE(0:0/18:0), 15-keto prostaglandin F1α, LPE(P-16:0), LPE(P-16:0), and d-gluconic acid. 229 endogenous metabolites were selected which contributed the most to the separation of post– TOF group and pre– TOF group. 40 metabolites were down regulated and 189 metabolites were up regulated (Figure 5D). Result of metabolites ranking top in terms of FC was shown in Figure 5E, including TG(16:0_20:4_22:6), n(nlpha)-acetyl-epsilon-(2-propenal)lysine, theobromine, TXB2, CE(20:4), 1,7-dimethylxanthine, TG(18:1_18:1_22:6), LPE(0:0/20:5), LPC(O-14:1), LPC(O-14:1), TG(8:0_16:0_18:2), CerP(d18:1/20:3), TG(12:0_14:0_16:1), TG(8:0_16:0_18:1), TG(10:0_14:0_16:0), TG(8:0_14:0_18:2), 6-hydroxynicotinic acid, TG(8:0_14:0_16:0), phenoxyacetic acid. Figure 5F showed the significant DEMs with high VIP scores, including CerP(d18:1/20:3), SM(d18:0/22:0), PC(24:0_18:1), LPC(20:4/0:0), PE(18:0_24:3), LPC(24:0/0:0), n(alpha)-acetyl- epsilon-(2-propenal)lysine, LPC(20:0/0:0), PE(16:0_22:5), PE (16:0_22:5), carnitine C8-OH, PI(18:0_22:4), LPE(0:0/18:2), PE(19:0_20:4), LPS(20:4/0:0), PC(18:0_24:3), PE(24:0_16:1), SM(d18:1/24:0), PC(16:0_20:3), PC(16:0_20:3).

### 3.5. Functional annotation and enrichment

**FIGURE 6:**
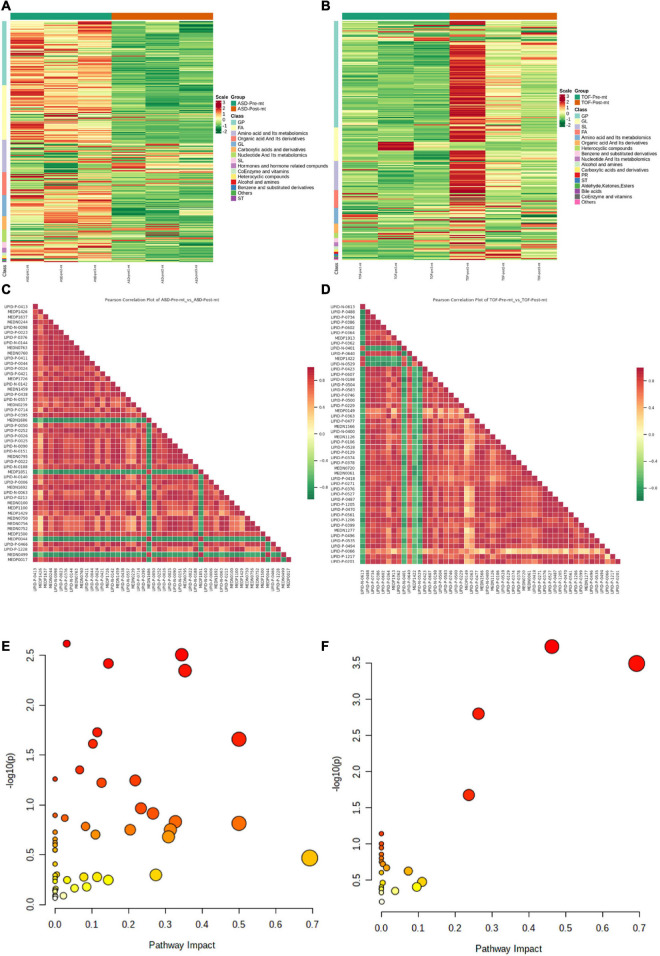
Differential metabolite enrichment analysis. **(A)** Heatmap of differential metabolites in atrial septal defect (ASD) samples. **(B)** Heatmap of differential metabolites in tetralogy of fallot (TOF) samples. **(C)** Heatmap of differential metabolite correlations in ASD samples. **(D)** Heatmap of differential metabolite correlations in TOF samples. **(E)** Metabolic pathways of ASD. **(F)** Metabolic pathways of TOF.

The expression level of DEMs of ASD groups and TOF groups were shown in Figures 6A, B (Supplementary Tables 2, 3), respectively. Correlations of DEMs of pre-ASD and post-ASD groups, pre-TOF and post-TOF groups were shown in Figures 6C, D, respectively, and most DEMs were positively correlated. Figures 6E, F shown the annotation results of the DEMs in pre-ASD and post-ASD groups and the DEMs in pre-TOF and post-TOF groups were classified based on metabolic pathway. Here, pathways with significant enrichment (impact > 0.1) in TOF were caffeine metabolism (impact = 0.69), sphingolipid metabolism (impact = 0.46), glycerophospholipid metabolism (impact = 0.26), tryptophan metabolism (impact = 0.24), galactose metabolism (impact = 0.11). Pathways with significant enrichment (impact > 0.1) in ASD were caffeine metabolism (impact = 0.69), D-Glutamine and D-glutamate metabolism (impact = 0.5), riboflavin metabolism (impact = 0.5), alanine, aspartate and glutamate metabolism (impact = 0.35), histidine metabolism (impact = 0.34), cysteine and methionine metabolism (impact = 0.33), arachidonic acid metabolism (impact = 0.31), sphingolipid metabolism (impact = 0.31), tryptophan metabolism (impact = 0.27), nicotinate and nicotinamide metabolism (impact = 0.27), arginine biosynthesis (impact = 0.23), pentose phosphate pathway (impact = 0.22), glycerophospholipid metabolism (impact = 0.20), galactose metabolism (impact = 0.14), ether lipid metabolism (impact = 0.14), amino sugar and nucleotide sugar metabolism (impact = 0.13), pyrimidine metabolism (impact = 0.11), terpenoid backbone biosynthesis (impact = 0.11), arginine and proline metabolism (impact = 0.11), glutathione metabolism (impact = 0.10).

## 4. Discussion

In general, we found that patients with TOF and ASD have metabonomic characteristics related to energy metabolism, which indicates that congenital heart disease may affect the long-term metabolic health of patients. Energy is the basis of physical activity, basic metabolism and growth. Energy disorders are a major factor limiting growth, motor development, cognition (22). The risk of energy imbalance is more likely to occur in infants with coronary artery disease. Most infants with coronary artery disease are born at normal gestational age and weight, but have nutrition and growth problems in early infancy (23). TOF is a CHD characterized by hypoxemia. The possible pathogenesis may be related to the following facts: chronic fetal hypoxia changes the expression of heart genes, accelerates the withdrawal of preexisting cardiomyocytes from the cell cycle, increases cardiomyocyte apoptosis, and reduces the number of cardiomyocytes (24). Hypoxemia leads to insufficient myocardial oxygen supply, impaired mitochondrial function, energy generation disorder, decreased ATP production and energy metabolism disorder, which weaken myocardial contractility; when hypoxia occurs, the heart rate increases and the demand for oxygen increases, which aggravates myocardial hypoxia. ASD is a birth defect caused by abnormal cardiovascular development in embryonic stage. The ultrastructural changes of ASD myocardium are usually compensatory hypertrophy and degenerative changes. In the early stage of cardiac hypertrophy, the volume of mitochondria increased competitively, but in the late stage, its volume decreased. During myocardial ischemia, the ability of mitochondria to absorb and store Ca^2+^changes, the dysfunction of mitochondrial respiration and oxidative phosphorylation, the change of outer membrane permeability, affects the process of tricarboxylic acid circulation and oxidative phosphorylation, the increase of lactic acid production caused by anaerobic digestion, and the decrease of ATP production are important reasons for the abnormal contractility of myocardial cells in ASD patients.

Through the transcriptome and metabonomics, it is suggested that energy metabolism was an important metabolic change in the development of TOF and ASD. Most metabolic pathways obtained through GSEA enrichment analysis are energy metabolism related pathways, such as regulation of triglyceride metabolic process, positive regulation of triglyceride metabolic process, fructose metabolic process, aspartic acid metabolic process, sucrose metabolic process, energy storage metabolic process, pyruvate metabolic process and organic acid metabolic process. Figure 3A shows that regulation of triglyceride metabolic process plays an important role. AADAC, THRSP and C3 are the genes with high variation rate in this pathway. Previous studies have shown that high triglycerides are closely related to ischemic heart disease, incidence rate of myocardial infarction and all-cause mortality. Triglyceride is an effective energy donor. Abnormal changes of triglyceride metabolism also play an important role in the pathogenesis of heart disease.

As a new technology, metabonomics has been applied to cardiovascular disease research to study the metabolic network related to cardiovascular disease, so as to better understand its pathophysiological mechanism. This technique can measure multiple metabolites in biological fluids or tissues. The identified biomarkers can be used as diagnostic and prognostic tools and help guide specific interventions and treatment opportunities. In this study, there are 18 triglyceride metabolites in the differential metabolites pre-TOF and post-TOF, most of which have been increased. Among the differential metabolites pre-ASD and post-ASD, there were 11 triglyceride metabolites, all of which decreased. Triglycerides are mainly transported in the form of very low density lipoprotein (VLDL), chyle granules and their metabolic residues in plasma. The increase of chyle granules and excessive residual lipoprotein produced by VLDL hydrolysis are independent risk factors for cardiovascular disease (25). The increase of serum triglyceride level indicates that the content of residual lipoprotein increases, and the particle of residual lipoprotein is small, which can enter the artery intima, thus causing a variety of cardiovascular diseases. Several studies have shown that plasma triglyceride levels are associated with cardiovascular disease risk (26, 27). In this study, patients with TOF and ASD have triglyceride metabolism disorder, which indicates that regulating triglyceride may be an effective method to treat these two diseases. Specifically, in patients with TOF, a significant increase of 54 phosphatidylcholine (PC) and phosphatidylethanolamine (PE) metabolites were observed. During recent years, in ASD patients, phospholipid metabolism was important in regulation of lipoprotein, and it has been demonstrated in many knockout animal models and in dietary studies (28, 29). PC is not only the basic structure of cell membrane and organelle membrane, but also the main component of bile and lung surfactant, as well as the precursor of a variety of signaling molecules and inflammatory mediators. At the same time, it participates in signal transduction as a ligand of specific receptors and is necessary for lipoprotein assembly and secretion. It plays a vital in regulating cellular lipid homeostasis and metabolism. In addition, PC is very important for heart metabolism, participating in the energy generation of myocardial cells and playing a role in the heart. The changes of cardiac lipid homeostasis are not only the main metabolic characteristics of cardiac function, but also represent systemic effects such as inflammation and weakness. Wittenbecker et al. studied 331 patients with heart failure and 507 healthy volunteers and found that PC (32:0) was highly correlated with heart failure risk (30). Tang et al. found that the levels of PC, PE and sphingomyelin in erythrocytes of patients with heart failure decreased, while the levels of 7-ket ochratosterol, hemolytic PC, hemolytic PE, and ceramide increased. This may be associated with pathological process of heart failure (31).

In ASD patients, lysophosphatidylcholine (LPC) metabolites were down regulated, and in TOF patients, 14 LPC metabolites were up regulated. As a lipid biomolecule, LPC has received more and more attention in cardiovascular diseases. LPC cleaves PC by phospholipase A2 enzyme, or converts fatty acids into free cholesterol by lecithin cholesterol acyltransferase (32). Negative correlation between plasma LPC and cardiovascular disease (33).

The common metabolic pathways (impact > 0.1) between ASD and TOF were caffeine metabolism, sphingolipid metabolism, glycerophospholipid metabolism, tryptophan metabolism, galactose metabolism. Sphingolipid is an important phospholipid in cell membrane, which was important in maintaining cell membrane structure, forming lipid rafts, mediating plasma membrane dynamic balance, transportation and communication, etc. Sphingolipid is the second phospholipid in mammalian body, and 18% of phospholipid in serum is sphingomyelin. The abnormal metabolism of sphingomyelin is related to many cardiovascular diseases, such as atherosclerosis, coronary heart disease, etc. This study shows that sphingomyelin metabolism plays an important role in the pathogenesis of ASD and TOF. Glycerophospholipid is a kind of lipid with the largest content in the body. In addition to forming biofilm. Glycerophospholipid metabolism is one of the most important components to maintain homeostasis in the body. More and more evidences indicate that genes related to glycerophospholipid synthesis and remodeling are involved in cardiovascular diseases. The abnormal expression of glycerol phospholipid metabolism related regulatory genes often leads to changes in the properties and types of glycerol phospholipids in the body, thus participating in disease progression. This study found that regulating glycerophospholipid is an effective method to treat TOF and ASD.

Our research has certain limitations. The sample size of each group is small. The feasibility of using non-targeted metabonomics to determine biomarkers in patients with TOF and ASD. Because it is difficult to collect clinical samples, the number of samples included in this study is small. In future studies, we will expand the number of samples to further verify our research results. Research needs to be carried out in cooperation with other women’s and children’s medical centers to describe related metabolic pathways. In addition, the use of cardiotoxic drugs should be avoided in the treatment of TOF (34, 35).

## Data availability statement

The original contributions presented in this study are included in the article/Supplementary material, further inquiries can be directed to the corresponding authors.

## Ethics statement

In this study, from June 1, 2018 to January 1, 2020, 12 samples were collected from The Third Affiliated Hospital of Sun Yat-sen University, including 12 right atrial (RA) biopsies of 3 TOF patients and 3 ASD patients. All research protocols of this study were approved by the Ethics Committee of The Third Affiliated Hospital of Sun Yat-sen University (Approval number of the Ethics Committee: 202202-121-01). The patients/participants provided their written informed consent to participate in this study.

## Author contributions

LL and LH took responsibility for all aspects of the reliability and freedom from bias of the data presented and their discussed interpretations and drafted the manuscript. FZ, LY, and JH took responsibility for the statistical analyses and interpretation of the data. SH and LM took responsibility for the full text evaluation and guidance and performed the final approval of the version to be submitted. All authors read and approved the final manuscript.

## Footnotes

1
http://www.gsea-msigdb.org/gsea/msigdb/collections.jsp

2
https://hmdb.ca/

3
https://www.metaboanalyst.ca/
